# Acute Leukemia in Children

**DOI:** 10.1155/2015/787194

**Published:** 2015-05-20

**Authors:** Juan Manuel Mejía-Aranguré, Richard J. Q. McNally

**Affiliations:** ^1^Unit of Clinical Epidemiology, Pediatrics Hospital, National Medical Center “Century XXI”, Mexican Institute of Social Security (IMSS) and Health Research Coordination, 06720 Mexico, DF, Mexico; ^2^Institute of Health & Society, Newcastle University, Newcastle upon Tyne NE1 4LP, UK

Acute lymphoblastic leukemia (ALL) is the most common cancer during childhood. Nevertheless, in recent years there have been marked improvements in treatment and consequent survival. There are a number of patients that experience relapses and patients that cannot be cured. In this special issue different aspects relating to factors influencing the best response to treatment are described. M. Czogała et al. showed that plasma ammonia concentration reflexes L-asparaginase activity may be a useful tool for assessing treatment in children with ALL. In another study A. Medina-Sanson et al. identified the importance of a genetic polymorphism of deoxycytidine kinase and cytidine deaminase in assessing the toxicity by cytarabine in children with acute myeloid leukemia and found that this polymorphism might predict death in affected children.

A. Vilchis-Ordoñez et al. addressed a very interesting topic about subpopulations of leukemic cells that contribute to a proinflammatory microenvironment within B-ALL bone marrow that may cause a delay in the normal hematopoiesis.

Two studies assessed the participation of environmental risk factors in the development of acute leukemia. A. Morales-Sánchez et al. did not find that EBV, HCMV, HHV6, and HHV7 were related to the development of ALL. Furthermore, J. D. Ferreira et al. did not show that maternal alcohol consumption during pregnancy was associated with leukemia in young children.

Finally a study by V. C. Bekker-Méndez et al. demonstrated differences in the frequency of gene rearrangement between the Mexican population and populations from developed countries.

From these articles it is clear that throughout the world the survival of children with acute leukemia is a great concern, especially in less developed countries. It is very important to search for factors associated with treatment compliance in children and those factors that may be related to treatment response.

The microenvironment in the leukemic bone marrow is a very important factor in the etiology of acute leukemia. Recovery of the bone marrow during treatment is also very important and further studies are needed to address this important aspect.

The environmental etiology of acute leukemia is a challenge that still has not been resolved. To identify environmental risk factors is not easy. It is important to identify both the environmental risk factors and also the gene-environment interactions that increase the susceptibility to development of leukemia.

The presentation of acute kinds of leukemia in Hispanic children is different from other populations; notably clinical features, the age incidence peak, and molecular factors are different.

In this special issue the complexity of the molecular and clinical epidemiology of acute leukemia in children has been shown. There are countries where success has been clearly demonstrated, but in other countries this is not apparent. This disease kills more children in developed countries and some emerging economies. There is a need to strengthen the research groups that are working to identify the factors that affect the adherence to treatment and the response to treatment. However, there is also a need for integration with those research groups that are working on the etiology of leukemia, especially including epidemiology and cellular and molecular biology.

We need to understand more about leukemia, in order to be able to cure and prevent it. The challenge continues because it is not a dream to think that one day almost all kinds of leukemia in children could be cured and that one day this disease could be prevented.

*Juan Manuel Mejía-Aranguré* *Richard J. Q. McNally*

